# No Fine-Tuning, No Cry: Robust SVD for Compressing Deep Networks

**DOI:** 10.3390/s21165599

**Published:** 2021-08-19

**Authors:** Murad Tukan, Alaa Maalouf, Matan Weksler, Dan Feldman

**Affiliations:** 1 The Robotics and Big Data Lab, Department of Computer Science, University of Haifa, Haifa 3498838, Israel; amaalouf@campus.haifa.ac.il (A.M.); dfeldman@univ.haifa.ac.il (D.F.); 2 Samsung Research Israel, Herzliya 4659071, Israel; m.weksler@samsung.com

**Keywords:** matrix factorization, neural networks compression, robust low rank approximation, Löwner ellipsoid

## Abstract

A common technique for compressing a neural network is to compute the *k*-rank $\ell_{2}$ approximation $A_{k}$ of the matrix $A\in R^{n\times d}$ via SVD that corresponds to a fully connected layer (or embedding layer). Here, *d* is the number of input neurons in the layer, *n* is the number in the next one, and $A_{k}$ is stored in O((n+d)k) memory instead of O(nd). Then, a fine-tuning step is used to improve this initial compression. However, end users may not have the required computation resources, time, or budget to run this fine-tuning stage. Furthermore, the original training set may not be available. In this paper, we provide an algorithm for compressing neural networks using a similar initial compression time (to common techniques) but without the fine-tuning step. The main idea is replacing the *k*-rank $\ell_{2}$ approximation with $\ell_{p}$, for p∈[1,2], which is known to be less sensitive to outliers but much harder to compute. Our main technical result is a practical and provable approximation algorithm to compute it for any p≥1, based on modern techniques in computational geometry. Extensive experimental results on the GLUE benchmark for compressing the networks BERT, DistilBERT, XLNet, and RoBERTa confirm this theoretical advantage.

## 1. Introduction

Deep learning revolutionized machine learning by improving the accuracy by dozens of percents for fundamental tasks in natural language processing (NLP), speech/image recognition, etc. One of the disadvantages of deep learning is that in many cases, the classifier is extremely large compared to classical machine learning models. A large network usually requires expensive and stronger resources due to: (1) slower classification time, which may be a serious limitation, especially in real-time systems such as autonomous cars or real-time text/speech translations; (2) a large memory requirement, which makes it infeasible to store the network on RAM or on a device such as IoT/smartphones; and (3) high energy consumption which is related to the CPU/GPU time of each classification and requires larger batteries with shorter lifespan.

**Pipeline of network compression.** Given training data *P*, a common pipeline to obtain a compressed network consists of the following stages:

(i) Train a network *N* based on the training set *P*, starting from an initial random network.
(ii) Compress the network N to a small network $\tilde{N}$. The input *P* may be not involved in this stage.
(iii) Fine-tune the weights of $\tilde{N}$ by training it on *P*. This step aims to improve the accuracy of the network $\tilde{N}$ but does not change its size.

In this paper, our goal is to improve the compression step (ii) in order to avoid the fine-tuning step (iii) via suggesting a better and more robust compressing scheme. We suggest a novel low rank factorization technique for compressing an embedding layer of a given NLP model. This is motivated by the fact that in many networks, the embedding layer accounts for 20–40% of the network size. Indeed, the results are easily extended to fully connected layers.

### 1.1. Embedding Matrix

One of the most common approaches for compressing neural networks is to treat a layer in the network as a matrix operation and then to approximate this matrix by its compressed version. This is especially relevant in a fully connected layer. Specifically, in word embedding, this layer is called the embedding layer, which is defined by the following matrix.

The input of the embedding layer consists of *d* input neurons, and the output has *n* neurons. The nd edges between these layers define a matrix $A\in {\mathbb{R}}^{n\times d}$. Here, the entry $A_{i,j}$ in the *i*th row and *j*th column of *A* is equal to the weight of the edge between the *j*th input neuron to the *i*th output neuron. Suppose that a test sample (vector) $x\in {\mathbb{R}}^{d}$ is received as an input. The corresponding output *n*-dimensional vector is thus y=Ax. To simply, a column from *A* during training is read, and a standard vector *x* (a column of the identity matrix) is used and is called a one-hot vector.

**$\ell_{2}$ *k*-rank approximation.** One of the natural and common matrix approximations, including in the context of network compression, is the $\ell_{2}$ *k*-rank approximation (see, e.g., [1,2] and references therein). This is the matrix which minimizes the Frobenius norm, i.e., the sum of squared distances ${∥A-A_{k}∥}_{F}^{2}:=\sum_{i=1}^{n}{∥A^{\left(i\right)}-A_{k}^{\left(i\right)}∥}_{2}^{2}$ between the *i*th row $A^{\left(i\right)}$ in *A* and its corresponding row $A_{k}^{\left(i\right)}$ in $A_{k}$, over every rank *k* matrix $A_{k}$. It can be easily computed via the singular value decomposition (SVD) in $O(\min\left\{nd^{2},dn^{2}\right\})$ time. Although $A_{k}$ has the same size as *A*, due to its low rank, it can be factorized as $A_{k}=UW$, where $U\in {\mathbb{R}}^{n\times k}$ and $W\in {\mathbb{R}}^{k\times d}$. We can then replace the original embedding layer that corresponds to *A* by a pair of layers that correspond to *U* and *W*, which can be stored using O(k(n+d)) memory, compared to the O(nd) entries in *A*. Moreover, the computation of the output $y_{k}:=A_{k}x$ takes O(k(n+d)) time, compared to the O(nd) time that it takes to compute Ax.

**Handling other linear layers.** The rank approximation technique can be also applied to a fully connected layer, where an activation function $f:{\mathbb{R}}^{n}\to \mathbb{R}$ is applied on the output Ax or each of its coordinates (as Relu) to obtain f(Ax). By approximating *A*, in a sense, f(Ax) is also approximated by $f\left(A_{k}x\right)$. Then, $A_{k}$ is replaced by two smaller layers U,W, as explained above. Furthermore, it is known that convolutional layers (tensors) can be viewed as fully connected layers (matrix multiplication) applied to reshaped volumes of the input. Then, one can approximate the convolutional weights by approximating its corresponding weight matrix. Hence, the rank approximation technique can be also applied to a convolutional layer.

### 1.2. Motivation

In what follows, we explain the main motivation of this paper, which in sum, aims to eliminate the need for the fine-tuning step due to the reasons explained in Section 1.2.1. We also discuss the weaknesses of the known SVD factorization in Section 1.2.2, which in turn, give rise to the motivation behind our approach discussed in Section 1.2.3.

#### 1.2.1. Fine-Tuning

The layers that correspond to the matrices *U* and *W* above are usually used only as initial seeds for a training process that is called fine-tuning, where the aim is to improve the initial results. Here, the training data are fed into the network, and as opposed to the $\ell_{2}$ error, the error is measured with respect to the final classification, i.e., in the fine-tuning step, the compressed network $\tilde{N}$ is trained using the input *P*, similar to Step (i). The goal of this step is to improve the accuracy of the compressed network without increasing its size. Hence, the structure of the data remains the same, but the edges are updated in each iteration.

**To be or not to be fine-tuned?** Fine-tuning is a necessary step to recover the generalization ability damaged by the model compression. Despite its widespread use, fine-tuning is vaguely understood, e.g., what fraction of the pre-trained weights are actually changing and why? [3].

In many cases, the fine-tuning cannot be applied:

1. The original (large) training set is not necessarily available for us (e.g., for sake of data privacy) to apply the fine-tuning after compressing the model.
2. For large datasets and complex tasks, the fine-tuning process takes a very long time and requires strong resources [4,5], even on the pruned networks. Hence, due to the limited computational power (and/or insufficient training time) of the end user device (e.g., smartphone, IoT), fine-tuning is not a viable choice.
3. In the context of NLP, it is common to learn representations of natural language [6,7,8,9] via full-network pre-training followed by fine-tuning on a small dataset for the sake of learning a new task [10,11,12]. However such pre-trained models are very large. Thus, a natural coping mechanism would involve compression before the fine-tuning. After the compression, the model suffers from loss in its original learning capability, and unfortunately, the fine-tuning process is not sufficient to both retain the model’s quality and make the network learn a new task, since we may not be able to obtain enough tagged information that we can rely on to perform meaningful training from scratch, e.g., when compressing the embedding layer, we may lose the richness of the vocabulary, as it is responsible for representing each word from a vocabulary by a vector that reflects its semantic and syntactic information which can be extracted from the language.

Hence, some have attempted to prune each layer independently, by which a fine-tuning process can be done with a small number of epochs to avoid the excessive computational power required by the fine-tuning process [5]. Finally, it is worth mentioning that the fine-tuned parameters are not constrained to share any components with the pre-trained weights and thus are equally expensive to store and to compute per iteration [13].

In this paper, we replace the go-to method for compression models using matrix factorization by a more robust low rank approximation scheme, where the emphasis here is that the learning capability of the model after the compression is less affected.

#### 1.2.2. Should We Use SVD?

Training the network and compressing it are natural steps. However, it is not clear that the last fine-tuning step, which may be a serious time consumer, is necessary. The goal of this work is to remove this step by improving the previous (compression) step via more involved algorithms that provably approximate the more robust $\ell_{p}$ rank approximation. We begin with geometric intuition.

**The geometry behind SVD.** Geometrically, each row of *A* corresponds to a *d*-dimensional vector (point) in ${\mathbb{R}}^{d}$, and the corresponding row in $A_{k}$ is its projection on a *k*-dimensional subspace of ${\mathbb{R}}^{d}$. This subspace (which is the column space of *U*) minimizes the sum of squared distances to the rows of *A* over every *k*-subspace in ${\mathbb{R}}^{d}$.

**Figure 1:**
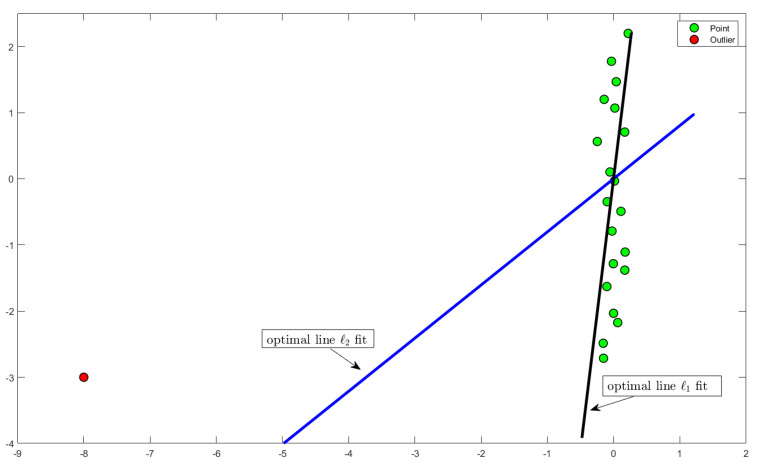
$\ell_{1}$-low rank approximation versus $\ell_{2}$-low rank approximation. Since the norm of a vector increases as the base of the norm decreases, the optimization problem becomes less susceptible towards outliers in the data as presented above.

Statistically, if these *n* points were generated by adding a Gaussian noise to a set of *n* points on a *k*-dimensional subspace, then it is easy to prove that most likely (in the sense of maximum-likelihood) this subspace is *U*. The disadvantage of $\ell_{2}$ *k*-rank approximation is that it is optimal under the above statistical assumption, which rarely seems to be the case for most applications. In particular, minimizing the sum of squared distances is heavily sensitive to outliers [14] (see Figure 1). As explained in [15], this is the result of squaring each term, which effectively weights large errors more heavily than small ones.

This undesirable property, in many applications, has led researchers to use alternatives such as the mean absolute error (MAD), which minimizes the $\ell_{1}$ (sum of distances) of the error vector. For example, compressed sensing [16] uses $\ell_{1}$ approximation as its main tool to clean corrupted data [17] as well as to obtain sparsified embeddings with provable guarantees as explained, e.g., in [18].

In machine learning, the $\ell_{1}$-approximation replaces or is combined with the $\ell_{2}$ approximation. Examples in scikit-learn include lasso regression, elastic-nets, or MAD error in decision trees [19].

#### 1.2.3. Novel Approach and Its Challenges

**Novel approach: deep learning meets subspace approximation.** We suggest generalizing the above $\ell_{2}$ approximation to $\ell_{1}$ *k*-rank approximation, or even $\ell_{p}$ approximation for more general p<2. Geometrically, we wish to compute the *k*-subspace that minimizes the sum of *p*th power of the distances to the given set of *n* points. This should result in more accurate compressed networks that are more robust to outliers and classification mistakes.

Unlike the case p=2, which was solved more than a century ago [20] via SVD and its variants, the $\ell_{p}$ low rank approximation was recently proved to be NP-hard even to approximate up to a factor of 1+1/poly(d) (recall that *d* is the number of columns of *A* above) for p∈[1,2) [21] and even for general (including constant) values of *p* (see Section 2). In the most recent decade, there was great progress in this area; however, the algorithms were either based on ad hoc heuristics with no provable bounds or impractical, i.e., their running time is exponential in *k* [21,22], and their efficiency in practice is not clear. Indeed, we could not find implementations of such provable algorithms.

This motivates the questions that are answered affirmably in this paper: (i) Can we efficiently compute the corresponding $\ell_{p}$ *k*-rank approximation matrix $A_{k}$, similar to SVD? (ii) Can we remove the fine-tuning step by using the $\ell_{p}$ low rank approximation, while scarifying only a small decrease in the accuracy of the compressed network? (iii) Can we obtain smaller networks with higher accuracy (without fine-tuning) by minimizing the sum of non-squared errors, or any other power p≠2 of distances, instead of the $\ell_{2}$ *k*-rank approximation via SVD?

### 1.3. Our Contribution

We answer these questions by suggesting the following contributions:

1. A new approach for compressing networks based on $\ell_{p}$ *k*-rank approximation instead of $\ell_{2}$, for p∈[1,∞). The main motivation is the robustness to outliers and noise, which is supported by many theoretical justifications.
2. Provable algorithms for computing this $\ell_{p}$ low rank approximation of every n×d matrix *A*. The deterministic version takes time $O(nd^{3}\log n)$, and the randomized version takes O(ndlogn). The approximation factor depends polynomially on *d*, is independent of *n* for the deterministic version, and is only poly-logarithmic in *n* for the randomized version.
3. Experimental results confirming that our approach significantly improves existing results when the fine-tuning step is removed from the pipeline upon using SVD (see Section 5).
4. Full open source code is provided [23].

Our results are based on a novel combination of modern techniques in computational geometry and applied deep learning. We expect that future papers will extend this approach (see Section 7).

To obtain efficient implementations with provable guarantees, we suggest a leeway by allowing the approximation factor to be larger than *k*, instead of aiming for (1+ε)-approximation (PTAS). In practice, this worst-case bound seems to be too pessimistic, and the empirical approximation error in our experiments is much smaller. This phenomenon is common in approximation algorithms, especially in deep learning, when the dataset has a lot of structure and is very different from synthetic worse-case artificial examples. The main mathematical tool that we use is the Löwner ellipsoid, which generalizes the SVD case to general $\ell_{p}$ cases, inspired by many papers in the related work below.

**To be part and not apart.** Our technique can be combined with previous known works to obtain better compression. For example, DistilBERT [24] is based on knowledge distillation, and it reduces the size of the BERT [12] model by 40%, while maintaining 97% of its language understanding capabilities and being 60% faster. However, this result does not use low rank factorization to compress the embedding layer. We further compressed DistilBERT and achieved better accuracy than SVD.

## 2. Related Work

In the context of training giant models, some interesting approaches were suggested to reduce the memory requirement, e.g., [25,26]. However, those methods reduced the memory requirement at the cost of speed/performance. Later, [27] proposed a way to train large models based on parallelization. Here, the model size and evaluation speed are also still an obstacle. Hence, many papers were dedicated to the purpose of compressing neural networks in the field of NLP. These papers are based on different approaches such as pruning [28,29,30,31,32], quantization [33,34], knowledge distillation [24,35,36,37,38,39,40,41], weight sharing [42], and low rank factorization [42,43,44] (see the example table in [45] for compressing the BERT model). There is no convention for which approach from the above should be used. However, recent works, e.g., [42], showed that combining such approaches yields good results.

**Subspace approximation.** The $\ell_{2}$ *k*-rank approximation can be solved easily in $\min\left\{nd^{2},d^{2}n\right\}$ time, while a (1+ε) approximation can be computed deterministically in $nd{\left(k/\varepsilon \right)}^{O(1)}$ time [46] for every ε>0, and a randomized version takes $O\mathrm{nnz}(A)+\left(n+d\right)\cdot {\left(k/\varepsilon \right)}^{O(1)}$ time, where nnz(*A*) is the number of non-zero entries in *A* [47,48,49]. These and many of the following results are summarized in the seminal work of [21]. However, for p≠2, even computing a multiplicative (1+ε)-approximation is NP-hard when *k* is part of the input [21]. Nevertheless, it is an active research area, where techniques from computational geometry are frequently used. The case p≥1 was introduced in the theory community by [50], and earlier, the case p=1 was introduced in the machine learning community by [51]. In [50], a randomized algorithm for any p≥1 that runs in time $nd2^{{\left(k/\varepsilon \right)}^{O(p)}}$ was suggested. The state of the art for p∈[1,2) in [21] takes $O\left(\mathrm{nnz}(A)+\left(n+d\right){\left(k/\varepsilon \right)}^{O(1)}+2^{\left({\left(k/\varepsilon \right)}^{O(1)}\right)}\right)$ time.

Approximation algorithms for the $\ell_{p}$ low rank approximation were suggested in [52] for any p≥1, which we also handle. Although the obtained approximation, in some cases, is smaller than the approximation achieved in this paper, the running time in most cases (depending on k) is much larger than that of ours.

Regardless of the approximation, [52] suggests a polynomial time algorithm (one of many) as long as $k\in \Theta \left(\frac{\log n}{\log\log n}\right)$. Similar to the discussion in [52], our $\ell_{1}$ low rank approximation allows us to recover an approximating matrix of any chosen rank, while the robust PCA [53] returns some matrix of unknown rank. Although variants of robust PCA have been proposed to force the output rank to be a given value [54,55], these variants make assumptions about the input matrix, whereas our results do not. The time complexity for p=1 was improved in [56] to $nd{\left(k/\varepsilon \right)}^{O(1)}+\left(n+d\right)2^{{\left(k/\varepsilon \right)}^{O(1)}}$, and later, for general *p* to $nd{\left(k/\varepsilon \right)}^{O(1)}+2^{{\left(k/\varepsilon \right)}^{O(p)}}$ [22]. The latter work, together with [57], also gives a *coreset* for subspace approximation, i.e., a way of reducing the number of rows of *A* so as to obtain a matrix $A^{\prime }$ such that the cost of fitting the rows of $A^{\prime }$ to any *k*-dimensional subspace *F* is within a 1+ε factor of the cost of fitting the rows of *A* to *F*; for p=2, such coresets were known [47,58,59,60] and can be computed exactly (ε=0) [61,62].

**Efficient approximations.** The exponential dependency on *k* and hardness results may explain why we could not find (even inefficient) open or closed code implementations on the web. To our knowledge, it is an open problem to compute larger factor approximations (ε∈O(1)) in a time polynomial in *k*, even in theory. The goal of this paper is to provide such a provable approximation in time that is near-linear in *n* with practical implementation and to demonstrate our usefulness in compressed networks.

## 3. Method

**Notations.** For a pair of integers n,d≥1, we denote by ${\mathbb{R}}^{n\times d}$ the set of all n×d real matrices, by $I_{d}\in {\left\{0,1\right\}}^{d\times d}$ the identity matrix, and [n]={1,⋯,n}. For a vector $x\in {\mathbb{R}}^{d}$, a matrix $A\in {\mathbb{R}}^{n\times d}$, and a real number p>0, the *p*th norm of *x* is defined as ${∥x∥}_{p}={\left(\sum_{i=1}^{d}{\left|x_{i}\right|}^{p}\right)}^{1/p}$, and the $\ell_{p}$ entry-wise norm of *A* is defined as ${∥A∥}_{p,p}={\left(\sum_{i=1}^{d}{∥Ae_{i}∥}_{p}^{p}\right)}^{1/p}$, where $e_{i}\in {\left\{0,1\right\}}^{d}$ is a vector whose *i*th entry is 1 and 0 elsewhere. We say that the columns of a matrix $A\in {\mathbb{R}}^{n\times d}$ (where n≥d) are orthogonal if $A^{T}A=I_{d}$. In addition, a matrix $F\in {\mathbb{R}}^{d\times d}$ is called positive definite matrix if *F* is a symmetric matrix, and for every $x\in {\mathbb{R}}^{d}$ such that ${∥x∥}_{2}>0$, we have $x^{T}Fx>0$. Furthermore, we say that a set $L\subseteq {\mathbb{R}}^{d}$ is centrally symmetric if for every x∈L, it holds that −x∈L. Finally, a set $L\subseteq {\mathbb{R}}^{d}$ is called a convex set if for every x,y∈L and θ∈[0,1], θx+(1−θ)y∈L.

### 3.1. ${∥\cdot ∥}_{p}$-SVD Factorization and the Löwner Ellipsoid

In what follows, we intuitively and formally describe the tools that will be used in our approach. Definition 1 is based on Definition 4 in [63]. While the latter defines a generic factorization for a wide family of functions, Definition 1 focuses on our case, i.e., the function we wish to factorize is ${∥Ax∥}_{p}$ for any p≥1, where $A\in {\mathbb{R}}^{n\times d}$ is the input matrix, and *x* is any vector in ${\mathbb{R}}^{d}$.

**Definition** **1** (Variant of Definition 4 [63]). *Let $A\in {\mathbb{R}}^{n\times d}$ be a matrix of rank *d*, and let p≥1 be a real number. Suppose that there is a diagonal matrix $D\in {\left(0,\infty \right)}^{d\times d}$ of rank *d*, and an orthogonal matrix $V\in {\mathbb{R}}^{d\times d}$, such that for every $x\in {\mathbb{R}}^{d}$,*

$${∥DV^{T}x∥}_{2}^{p}\leq {∥Ax∥}_{p}^{p}\leq d^{\frac{p}{2}}{∥DV^{T}x∥}_{2}^{p}.$$

*Define $U=A{\left(DV^{T}\right)}^{-1}$. Then, $UDV^{T}=A$ is called the ${∥\cdot ∥}_{p}$-SVD of *A*.*

**Why ${∥\cdot ∥}_{p}$-SVD?** The idea behind using the ${∥\cdot ∥}_{p}$-SVD factorization of an input matrix *A* is that we obtain a way to approximate the span of the column space of $A\in {\mathbb{R}}^{n\times d}$. This allows us to approximate the dot product Ax for any $x\in {\mathbb{R}}^{d}$, which implies an approximation for the optimal solution of the $\ell_{p}$ low rank approximation problem.

For example, in the case of p=2, the ${∥\cdot ∥}_{2}$-SVD of a matrix $A\in {\mathbb{R}}^{n\times d}$ is equivalent to the known SVD factorization $A=UDV^{T}$. This holds due to the fact that the columns of the matrix *U* are orthogonal, and for every $x\in {\mathbb{R}}^{d}$, we have ${∥Ax∥}_{2}^{2}={∥UDV^{T}x∥}_{2}^{2}={∥DV^{T}x∥}_{2}^{2}.$ As for the general case of any p≥1, [63] showed that the ${∥\cdot ∥}_{p}$-SVD factorization always exists, and can be obtained using the *Löwner* ellipsoid.

**Theorem** **2** (Variant of Theorem III [64]). *Let $D\in {\left[0,\infty \right)}^{d\times d}$ be a diagonal matrix of full rank and an orthogonal matrix $V\in {\mathbb{R}}^{d\times d}$, and let E be an ellipsoid defined as $E=\left\{x\in {\mathbb{R}}^{d}|x^{T}VD^{T}DV^{T}x\leq 1\right\}.$*

*Let L be a centrally symmetric compact convex set. Then, there exists a unique ellipsoid E called the*Löwner ellipsoid*of L such that $1/\sqrt{d}E\subseteq L\subseteq E$, where $1/\sqrt{d}E=\left\{1/\sqrt{d}x|x\in E\right\}$.*

**Computing ${∥\cdot ∥}_{p}$-SVD via Löwner ellipsoid.** Intuitively speaking, for an input matrix $A\in {\mathbb{R}}^{n\times d}$, the ${∥\cdot ∥}_{p}$-SVD $A=UDV^{T}$ aims to bound from above and below the cost function ${∥Ax∥}_{p}^{p}$ for any $x\in {\mathbb{R}}^{d}$ by the term ${∥DV^{T}x∥}_{2}^{p}$. Since ${∥Ax∥}_{p}^{p}$ is a convex continuous function (for every $x\in {\mathbb{R}}^{d}$), the level set $L=\left\{x\in {\mathbb{R}}^{d}|{∥Ax∥}_{p}\leq 1\right\}$ is also convex. Having a convex set enables us to use the *Löwner ellipsoid*, which, in short, is the minimum volume enclosing ellipsoid of *L*. In addition, contracting the *Löwner ellipsoid* by $\sqrt{d}$ yields an inscribed ellipsoid in *L*. It turns out that D,V of the ${∥\cdot ∥}_{p}$-SVD represents the Löwner ellipsoid of *L* as follows: *D* is a diagonal matrix such that its diagonal entries contain the reciprocal values of the ellipsoid axis lengths, and *V* is an orthogonal matrix which is the basis of the same ellipsoid. Using the enclosing and inscribed ellipsoids (the Löwner ellipsoid and its contracted form) enables us to bound ${∥\cdot ∥}_{p}$ using the mahalonobis distance. Although in traditional *k*$\ell_{2}$-low rank factorization with respect to an input matrix $A\in {\mathbb{R}}^{n\times d}$, the optimal result is equal to the sum of the smallest d−k singular values, we generalize this concept to $\ell_{p}$-low rank factorization. Specifically, the singular values of *D* (the reciprocal values of the ellipsoid axis lengths) serve as a bound on the “$\ell_{p}$” singular values of *A*.

### 3.2. Additive Approximation for the $\ell_{p}$-Low Rank Factorization

In what follows, we show how to compute an approximated solution for the $\ell_{p}$-low rank factorization for any p≥1 (see Algorithm 1). This is based on the ${∥\cdot ∥}_{p}$-SVD factorization (see Definition 1).

**From ${∥\cdot ∥}_{p}$-SVD to $\ell_{p}$-low rank factorization.** For any k∈[d−1] and any matrix $A\in {\mathbb{R}}^{n\times d}$ of rank *d*, the $\ell_{p}$-low rank factorization problem aims to minimize ${∥A-AXX^{T}∥}_{p,p}^{p}$ over every matrix $X\in {\mathbb{R}}^{d\times k}$ whose columns are orthogonal. As a byproduct of the orthogonality of *X*, the problem above is equivalent to minimizing ${∥AYY^{T}∥}_{p,p}^{p}$ over every matrix $Y\in {\mathbb{R}}^{d\times \left(d-k\right)}$ whose columns are orthogonal such that $YY^{T}=I_{d}-XX^{T}$. By exploiting the definition of the entry-wise $\ell_{p}$ norm of $AYY^{T}$, we can use ${∥\cdot ∥}_{p}$-SVD to bound this term from above and below using the mahalonobis distance. Furthermore, we will show that by using the ${∥\cdot ∥}_{p}$-SVD, we can compute a matrix $A_{k}$ of rank *k* such that ${∥A-A_{k}∥}_{p,p}^{p}$ depends on the ellipsoid axis lengths (see Algorithm 1 and Theorem 5).

**Figure 2:**
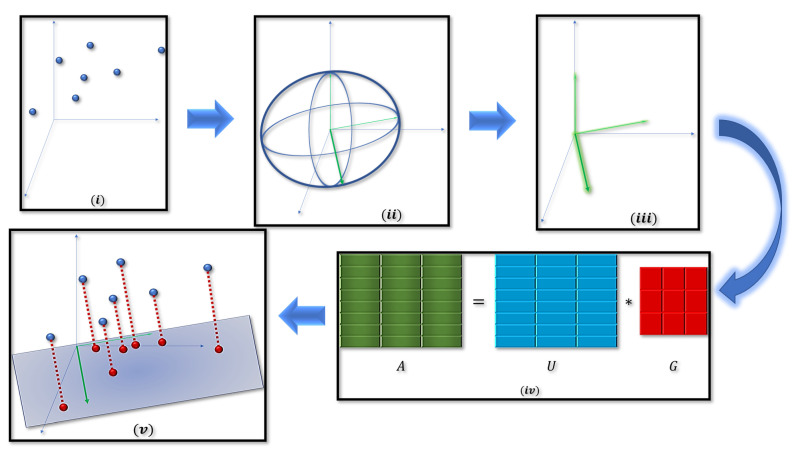
Illustration of our method. Given a matrix $A\in {\mathbb{R}}^{n\times d}$ whose rows are points in ${\mathbb{R}}^{d}$ (step (***i***)), we first compute the Löwner ellipsoid of $f\left(x\right){=∥Ax∥}_{p}$ for every $x\in {\mathbb{R}}^{d}$ (step (***ii***)). This enables us to encapsulate the geometrical properties of *f*. After computing the minimum volume enclosing ellipsoid, we focus on the ellipsoids’ axes which will form our matrix *G* (step (***iii***)). Due to the invertability of *G*, we can factorize *A* into a multiplication of two matrices, $U=AG^{-1}$ and *G* (step (***iv***)). Finally, we choose the longest *k* axes of the ellipsoid where these vectors will form a subspace on which the points will be projected on to form our low rank approximation as illustrated above (step (***v***), see red points).

**Overview of Algorithm 1.** Algorithm 1 receives as input a matrix $A\in {\mathbb{R}}^{n\times d}$ of rank *d*, a positive integer k∈[d−1], and a positive number p≥1 and outputs a matrix $A_{k}$ of rank *k*, which satisfies Theorem 5. At Line 1, we compute a pair of matrices $D,V\in {\mathbb{R}}^{d\times d}$ such that the ellipsoid $E:=\left\{x\in {\mathbb{R}}^{d}|x^{T}VD^{T}DV^{T}x\leq 1\right\}$ is the Löwner ellipsoid of $L:=\left\{x\in {\mathbb{R}}^{d}|{∥Ax∥}_{p}\leq 1\right\}$, where *D* is a diagonal matrix of rank *d*, and *V* is an orthogonal matrix; we refer the reader to the Appendix A for computing the Löwner ellipsoid. At Line 2, we compute the matrix *U* from the ${∥\cdot ∥}_{p}$-SVD of *A* (see Definition 1). At Lines 3–4, we set $D_{k}$ to be the diagonal matrix of d×d entries where the first *k* diagonal entries are identical to the first *k* diagonal entries of *D*, while the rest of the matrix is set to 0 (see Figure 2 for an illustrative description of our algorithm).
**Algorithm 1:***ℓ_ρ_*-LOW-RANK (*A*, *k*, *p*)
 **Input** **:**A matrix $A\in {\mathbb{R}}^{n\times d}$ of rank *d*, p≥1, a positive integer k∈[d−1], and a positive real number p≥1. **Output** **:**A matrix $U\in {\mathbb{R}}^{n\times d}$, a diagonal matrix $D_{k}\in {\left[0,\infty \right)}^{d\times d}$, an orthogonal matrix $V\in {\mathbb{R}}^{d\times d}$ where U,V are from the ${∥\cdot ∥}_{p}$-SVD of *A*, and a set of *d* positive real numbers $\left\{\sigma_{1},\ldots ,\sigma_{d}\right\}$.(D,V):=LÖWNER(A,p) //(See Algorithm A1 at the Appendix A$U:=A{\left(DV^{T}\right)}^{-1}$ //(computing *U* from the ${∥\cdot ∥}_{p}$-SVD of *A* with respect to the $\ell_{p}$-regression problem$\left\{\sigma_{1},\ldots ,\sigma_{d}\right\}:=$ the diagonal entries of *D*$D_{k}:=\mathrm{diag}\left(\sigma_{1},\ldots ,\sigma_{k},0,\ldots ,0\right)$ //(A diagonal matrix in ${\mathbb{R}}^{d\times d}$**return** $U,D_{k},V,\left\{\sigma_{1},\ldots ,\sigma_{d}\right\}$

## 4. Analysis

Some of the proofs in this section were moved into the Supplementary Material due to space limitations.

### 4.1. Deterministic Result

In what follows, we present our deterministic solution for the $\ell_{p}$-low rank factorization problem.

**Claim** **3.** *Let* $D\in {\left[0,\infty \right)}^{d\times d}$ *be a diagonal matrix of rank* *d* *, and let* σ>0 *be the lowest singular value of* *D* *. Then, for every unit vector* $x\in {\mathbb{R}}^{d}$ *,* ${∥Dx∥}_{2}\geq \sigma .$

**Proof.**  Let $x\in {\mathbb{R}}^{d}$ be a unit vector, and for every i∈[d], let $D_{i,i}$ denote the *i*th diagonal entry of *D*, and $x_{i}$ denotes the *i*th entry of *x*. Observe that

$$\begin{array}{c} {∥Dx∥}_{2}={\left(\sum_{i=1}^{d}{\left|D_{i,i}x_{i}\right|}^{2}\right)}^{\frac{1}{2}}\geq {\left(\sum_{i=1}^{d}{\left|\sigma x_{i}\right|}^{2}\right)}^{\frac{1}{2}}=\sigma {∥x∥}_{2}=\sigma , \end{array}$$

where the first equality follows from the definition of norm, the inequality holds by definition of σ, and the last equality holds since *x* is a unit vector.    □

**Lemma** **4** (Special case of Lemma 15 [63] *Let $A\in {\mathbb{R}}^{n\times d}$ be a matrix of full rank, p≥1. Then, there exist a diagonal matrix $D\in {\left[0,\infty \right)}^{d\times d}$ of full rank and an orthogonal matrix $V\in {\mathbb{R}}^{d\times d}$ such that for every $x\in {\mathbb{R}}^{d}$,*

(1) $${∥DV^{T}x∥}_{2}^{p}\leq {∥Ax∥}_{p}^{p}\leq d^{\frac{p}{2}}{∥DV^{T}x∥}_{2}^{p}$$

**Proof.**  First, let $L=\{\tilde{x}\in {\mathbb{R}}^{d}|{A\tilde{x}}_{p}^{p}\leq 1\}$, and put $x\in {\mathbb{R}}^{d}$. Observe that (i) since p≥1, the term ${∥A\tilde{x}∥}_{p}$ is a convex function for every $\tilde{x}\in {\mathbb{R}}^{d}$ which follows from properties of norm function. This means that the level set *L* is a convex set. In addition, (ii) by definition of *L*, it holds that for every $\tilde{x}\in L$, also $-\tilde{x}\in L$, which makes *L* a centrally symmetric set by definition. Note that (iii) since *A* is of full rank, then *L* spans ${\mathbb{R}}^{d}$.

Since properties (i)–(iii) hold, we obtain by Theorem 1 that there exists a diagonal matrix $D\in {\left[0,\infty \right)}^{d\times d}$ of full rank and an orthogonal matrix $V\in {\mathbb{R}}^{d}$ such that the set $E=\left\{\tilde{x}\in {\mathbb{R}}^{d}|{\tilde{x}}^{T}VD^{T}DV^{T}\tilde{x}\leq 1\right\}$ satisfies

(2) $$\frac{1}{\sqrt{d}}E\subseteq L\subseteq E.$$

**Proving the right hand side of Equation (1).** Let $y=\frac{1}{{∥DV^{T}x∥}_{2}}x$, and observe that

(3) $$\begin{array}{c} {∥Ax∥}_{p}^{p}={\left(\sqrt{d}{∥DV^{T}x∥}_{2}\right)}^{p}{∥\frac{1}{\sqrt{d}}Ay∥}_{p}^{p}\leq d^{\frac{p}{2}}{∥DV^{T}x∥}_{2}^{p}, \end{array}$$

where the equality follows from the definition of *y*, and the inequality holds since $\frac{1}{\sqrt{d}}y\in L$ follows from Equation (2).

**Proving the left hand side of Equation (1).** Since *L* spans ${\mathbb{R}}^{d}$, there then exists b>0 such that ${∥A\left(bx\right)∥}_{p}^{p}=1$. By Equation (2), bx∈E, which results in ${∥DV^{T}x∥}_{2}=\frac{1}{b}{∥DV^{T}bx∥}_{2}\leq \frac{1}{b}$. Thus,

(4) $${∥Ax∥}_{p}^{p}=\frac{1}{b^{p}}{∥Abx∥}_{p}^{p}=\frac{1}{b^{p}}\geq {∥DV^{T}x∥}_{2}^{p}$$

Since Equation (3) and Equation (4) hold for every $x\in {\mathbb{R}}^{d}$, Lemma 4 follows.    □

**Theorem** **5.** *Let $A\in {\mathbb{R}}^{n\times d}$ be real matrix, p≥1; k∈[d−1] be an integer; and ($U,D_{k},V,\sigma_{1},\ldots \sigma_{d}$) be the output of a call to ℓ_ρ_-*Low-rank*(A,k,p). Let $A_{k}=UD_{k}V^{T}$. Then,*

$$d\sigma_{d}^{p}\leq {∥A-A_{k}∥}_{p,p}^{p}\leq d^{1+\frac{p}{2}}\sigma_{k}^{p}.$$

**Proof.**  First, we assume that p≠2; otherwise, the ${∥\cdot ∥}_{2}$ factorization is the *SVD* factorization, and we obtain the optimal solution for the $\ell_{2}$ low rank approximation problem. For every i∈[d], let $e_{i}\in {\mathbb{R}}^{d}$ be a vector of zeros, except for its *i*th entry, where it is set to 1. Observe that ${∥A-A_{k}∥}_{p,p}^{p}=\sum_{i=1}^{d}{∥A-A_{k}e_{i}∥}_{p}^{p}=\sum_{i=1}^{d}{∥AI-{DV^{T}}^{-1}D_{k}V^{T}e_{i}∥}_{p}^{p}$, where the first equality holds by definition of ${∥\cdot ∥}_{p,p}^{p}$, and the second equality follows from the definition of $A_{k}$ (see Lines 3–4 of Algorithm 1).

Plugging A:=A, D:=D, V:=V, $x:=\left(I-{\left(DV^{T}\right)}^{-1}D_{k}V^{T}\right)e_{i}$ into Lemma 4 yields that for every i∈[d],

(5) $$\begin{array}{cc} {∥\left(D-D_{k}\right)V^{T}e_{i}∥}_{2}^{p} & \leq {∥\left(A-A_{k}\right)e_{i}∥}_{p,p}^{p}\\ \; & \leq d^{\frac{p}{2}}{∥\left(D-D_{k}\right)V^{T}e_{i}∥}_{2}^{p}. \end{array}$$

Observe that for every i∈[d],

(6) $$\begin{array}{c} {∥\left(D-D_{k}\right)V^{T}e_{i}∥}_{2}^{p}\leq {∥D-D_{k}∥}_{2}^{p}{∥V^{T}e_{i}∥}_{2}^{p}\leq {∥D-D_{k}∥}_{2}^{p}, \end{array}$$

where the first inequality holds by properties of the $\ell_{2}$ matrix induced norm, and the second inequality holds since *V* is an orthogonal matrix.

Since $V^{T}e_{i}$ is a unit vector,

(7) $${∥\left(D-D_{k}\right)V^{T}e_{i}∥}_{2}^{p}\geq \sigma_{d}^{p},$$

where the inequality holds by plugging $x:=V^{T}e_{i}$ and $D:=\left(D-D_{k}\right)$ into Claim 3.

In addition, we have that

(8) $$\sigma_{d}\leq {∥D-D_{k}∥}_{2}=\sigma_{k+1}$$

where both the inequality and equality hold since $\sigma_{d}$ is the lowest eigenvalue of *D*, *D* being a diagonal matrix.

By combining Equations (5)–(8), we obtain that for every i∈[d],

(9) $$\sigma_{d}^{p}\leq {∥\left(A-A_{k}\right)e_{i}∥}_{p}^{p}\leq d^{\frac{p}{2}}\sigma_{k+1}^{p}.$$

Theorem 5 follows by summing Equation (9) over every i∈[d].    □

**Note** that the set ${\left\{\sigma_{i}\right\}}_{i=1}^{d}$ denotes the reciprocal values of the ellipsoid *E* axis’s lengths, where *E* is the Löwner ellipsoid of $L=\left\{x\in {\mathbb{R}}^{d}|{∥Ax∥}_{p}\leq 1\right\}$. As discussed in the previous section, these values serve to bound the “ $\ell_{p}$ singular values of *A*”.

### 4.2. Randomized Result

In addition to our deterministic result, we also show how to support a randomized version that computes an approximation in a faster time, which relies on the following result of [65].

**Theorem** **6** (Variant of Theorem 10 [65]). *For any $A\in {\mathbb{R}}^{n\times d}$ of rank d and p≥1, one can compute an invertible matrix $R\in {\mathbb{R}}^{d\times d}$ and a matrix $U=AR^{-1}$ such that ${∥Rx∥}_{2}\leq {∥Ax∥}_{p}\leq d{\left(d^{3}+d^{2}\log n\right)}^{\left|1/p-1/2\right|}{∥Rx∥}_{2}$ holds with a probability of at least $1-\frac{1}{n}$, where R can be computed in time O(ndlogn).*

**Theorem** **7.** *Let $A\in {\mathbb{R}}^{n\times d}$ be real matrix, p≥1, and k∈[d−1] be an integer. There exists a randomized algorithm which, when given a matrix $A\in {\mathbb{R}}^{n\times d}$, k∈[d−1], in time O(ndlogn), returns $\left(U,D_{k},V,\left\{\sigma_{1},\ldots \sigma_{d}\right\}\right)$, such that*

$$d\sigma_{d}^{p}\leq {∥A-A_{k}∥}_{p,p}^{p}\leq d^{1+p}{\left(d^{3}+d^{2}\log n\right)}^{\left|1-p/2\right|}\sigma_{k+1}^{p},$$

*holds with a probability of at least $1-\frac{1}{n}$, where $A_{k}=UD_{k}V^{T}$.*

**Proof.**  The algorithm is described throughout the following proof. Let $R\in {\mathbb{R}}^{d\times d}$ be as defined in Theorem 6 when plugging A:=A into Theorem 6. Let $R=\tilde{U}DV^{T}$ be the *SVD* of *R*; $D_{k}\in {\left[0,\infty \right)}^{d\times d}$ be a diagonal matrix where its first *k* diagonal entries are identical to those of *D*, while the rest of the entries in $D_{k}$ are set to 0; and $\left\{\sigma_{1},\cdots ,\sigma_{d}\right\}$ be the set of singular values of *D*. Note that since for every $x\in {\mathbb{R}}^{d}$, by Theorem 6 it holds that

$${∥Rx∥}_{2}^{p}\leq {∥Ax∥}_{p}^{p}d^{p}{\left(d^{3}+d^{2}\log n\right)}^{\left|1-p/2\right|}{∥Rx∥}_{2}^{p}.$$

From here, similar to the proof of Theorem 5, we obtain that

$$d\sigma_{d}^{p}\leq {∥A-A_{k}∥}_{p,p}^{p}\leq d^{1+p}{\left(d^{3}+d^{2}\log n\right)}^{\left|1-p/2\right|}\sigma_{k+1}^{p}.$$

   □

**Remark** **8.** *Note that in our context of embedding layer compression, the corresponding embedding matrix A has more columns than rows. Regardless, our $\ell_{p}$ norm of any A−B such that A,B∈d×n enables us to have ${∥A-B∥}_{p,p}^{p}={∥A^{T}-B^{T}∥}_{p,p}^{p}.$ Hence, substituting $A:=A^{T}$ and $A_{k}:=A_{k}^{T}$ yields*

$$d\sigma_{d}^{p}\leq {∥A-A_{k}∥}_{p,p}^{p}\leq d^{1+\frac{p}{2}}\sigma_{k+1}^{p},$$

*for our deterministic results, and similarly, we can obtain this for our randomized result.*

## 5. Experimental Results

**The compressed networks.** We compress several frequently used NLP networks:

(i) BERT [12]: BERT is a bidirectional transformer pre-trained using a combination of masked language modeling objective and next sentence prediction on a large corpus comprising the Toronto Book Corpus and Wikipedia.
(ii) DistilBERT [24]: the DistilBERT model is smaller, faster, cheaper, and lighter than BERT. This model is a distilled version of BERT. It has 40% less parameters than bert-base-uncased and runs 60% faster, while preserving over 95% of BERT’s performances as measured on the GLUE language understanding benchmark [24].
(iii) XLNet [66]: XLnet is an extension of the Transformer-XL model [67] pre-trained using an autoregressive method to learn bidirectional contexts by maximizing the expected likelihood over all permutations of the input sequence factorization order.
(iv) RoBERTa [68]: RoBERTa modifies the key hyperparameters in BERT, including removing BERT’s next-sentence pre-training objective, and training with much larger mini-batches and learning rates. This allows RoBERTa to improve on the masked language modeling objective compared with BERT and leads to better downstream task performance.

See full details on the sizes of each network and their embedding layer before compression in Table 1.

**Implementation, Software, and Hardware.** All the experiments were conducted on an AWS p2.xlargs machine with 1 GPU NVIDIA K80, 4 vCPUs, and 61 RAM [GiB]. We implemented our suggested compression algorithm (Algorithm 1) in Python 3.8 using the Numpy library [69]. To build and train networks (i)–(iv), we used the suggested implementation in the Transformers https://github.com/huggingface/transformers (accessed on 15 July 2021) library from HuggingFace [70] (Transformers version 2.3 and PyTorch version 1.5.1 [71]). Before the compression, all the networks were fine-tuned on all the tasks from the GLUE benchmark to obtain almost the same accuracy results as reported in the original papers. Since we did not succeed in obtaining close accuracy on the tasks QQP and WNLI (with most of the network), we did not include results from them.

**Our compression**. We compress each embedding layer (matrix) of the reported networks by factorizing it into two smaller layers (matrices) as follows. For an embedding layer that is defined by a matrix $A\in {\mathbb{R}}^{n\times d}$, we compute the matrices $U,D_{k},V$ by a call to *ℓ_ρ_*-Low-rank A,k,1 (see Algorithm 1), where *k* is the low rank projection we wish to have. Observe, that the matrix $D_{k}$ is a diagonal matrix, and its last d−k columns are zero columns. We then compute a non-square diagonal matrix $D_{k}^{\prime }\in {\mathbb{R}}^{d\times k}$ that is the result of removing all the zero columns of $D_{k}$. Now, the $\ell_{1}$*k*-rank approximation of *A* can be factorized as $A_{k}=\left(U\sqrt{D_{k}^{\prime }}\right)\left({\sqrt{D_{k}^{\prime }}}^{T}V^{T}\right)$. Hence, we save the two matrices (layers): (i) $U\sqrt{D_{k}^{\prime }}$ of size n×k, and (ii) ${\sqrt{D_{k}^{\prime }}}^{T}V^{T}$ of size k×d. This yields two layers of a total size of nk+kd instead of a single embedding layer of a total size of nd.

**Figure 3:**
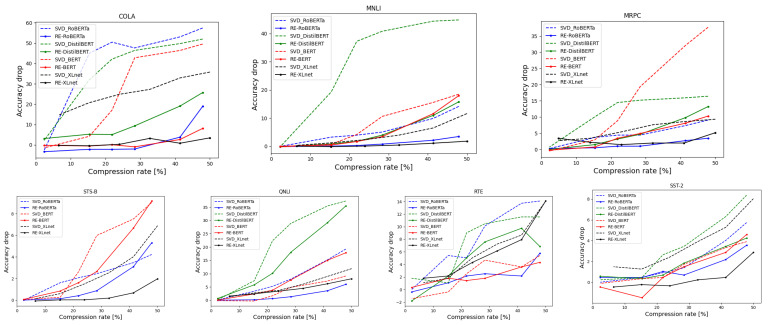
Here, we report the accuracy drop (additive error) as a function of the embedding layer’s compression rate on the networks (i)–(iv). We compare our results with SVD over several tasks from the GLUE benchmark. For a network “X”, our compressed version of it is called “RE-X”, e.g., RE-BERT and RE-XLNet.

**Reported results**. We report the test accuracy drop (relative error) on all the tasks from the GLUE benchmark [72] after compression for several compression rates:

1. In Figure 3, the *x*-axis is the compression rate of the embedding layer, and the *y*-axis is the accuracy drop (relative error) with respect to the original accuracy of the network. Each figure reports the results for a specific task from the GLUE benchmark on all the networks we compress. Here, all reported results are compared to the known $\ell_{2}$-factorization using SVD. In addition, in all the experiments, we do not fine-tune the model after compressing; this is to show the robustness and efficiency of our technique.
2. Table 2 suggests the best compressed networks in terms of accuracy vs size. For every network from (i)–(iv), we suggest a compressed version of it with a very small drop in the accuracy and sometimes with an improved accuracy. Given a network “X”, we call our compressed version of “X” “RE-X”, e.g., RE-BERT and RE-XLNet. The “RE” here stands for “Robust Embedding”.
3. Table 3 reports a comparison between our approach and different compressionmethods that do not require fine-tuning or any usage of the training data after compression:
  (i) SVD.
  (ii) L1PCA [73].
  (iii) Pruning [74].
  (iv) Random pruning.
  (v) Syn flow [75].

## 6. Discussion

It can be seen by Figure 3 that our approach is more robust than the traditional SVD. In most of the experiments, our suggested compression achieves better accuracy for the same compression rate compared to the traditional SVD. Mainly, we observed that our compression schemes shine when either vocabulary is rich (the number of subword units is large) or the model itself is small (excluding the embedding layer). Specifically speaking, in RoBERTa, our method achieves better results due to the fact that RoBERTa’s vocabulary is rich (i.e., 50 K subword units compared to the 30 K in BERT). This large vocabulary increases the probability of having outliers in it, which is the main justification for our approach. In DistilBERT, the network is highly efficient. This can lead to a sensitive snowball effect, i.e., the classification is highly affected by even the smallest errors caused by the compression of the embedding layer. Since SVD is sensitive to outliers and due to the fact that the network is highly sensitive to small errors, the existence of outliers highly affects the results. This phenomenon is illustrated throughout Figure 3. Here, our compression scheme outperforms the SVD due to its robustness against outliers, which, in turn, achieves smaller errors. As for XLNet, the model encodes the relative positional embedding, which, in short, represents an embedding of the relative positional distance between words. In our context, this means that having outliers highly affects the relative positional embedding, which, in turn, affects the classification accuracy. Hence, this explains why we outperform SVD. Since none of the above phenomena hold for BERT, this may explain why SVD sometimes achieves better results. However, across most tasks, our compression scheme is favorable upon SVD.

Finally, for some tasks at low compression rates, the accuracy has been improved (e.g., see task SST-2 at Figure 3 when compressing BERT). This may be due to the fact that at low compression rates, we remove the least necessary (redundant) dimensions. Thus, if these dimensions are actually unnecessary, by removing them, we obtain a generalized model which is capable of classifying better.

## 7. Conclusion and Future Work

We provided an algorithm that computes an approximation for $\ell_{p}$*k*-rank approximation, where p≥1. We then suggested a new approach for compressing networks based on *k*-rank $\ell_{p}$-approximation, where p∈[1,2] instead of $\ell_{2}$. The experimental results in Section 5 showed that our suggested algorithm overcomes the traditional $\ell_{2}$*k*-rank approximation and achieves higher accuracy for the same compression rate when there is no fine-tuning involved.

Future work includes: (1) Extending our approach to other factorization models, such as non-negative matrix approximation or dictionary learning; (2) experimental results on other benchmarks and other models; (3) suggesting algorithms for the $\ell_{p}$*k*-rank approximation for any p∈(0,1), while checking the practical contribution in compressing deep networks for this case; and (4) combining this result with other compression techniques to obtain a smaller network with higher accuracy.

## Figures and Tables

**Table 1:** The sizes of the networks BERT, RoBERTA, XLNet, and DistilBERT, and their embedding layers.

Model		Embedding Layer Size	Parameters
BERT	base	30,522 ×768	110 M
RoBERTa	base	50,265 ×768	125 M
XLNet	base	32,000 ×768	110 M
DistilBERT	base	30,522 ×768	66 M

**Table 2:** In the above, we present our compressed networks and their drop in accuracy based on the compression rate of the embedding layer. Specifically, each non-percentile value represents the accuracy drop achieved by our compressed model with respect to its original model (e.g., RE-RoBERTa is a compressed model of RoBERTa), while negative values present improvements in the accuracy upon the non-compressed version of the corresponding model. The last column is the average accuracy drop over all tested tasks. The “RE” here stands for “Robust Embedding”.

Model		Embedding Layer Compression Rate	MRPC	COLA	MNLI	SST-2	STS-B	QNLI	RTE	Avg.
RE-RoBERTa	base	15%	0.49	−2.16	0.01	0.045	0.013	0.018	1.08	−0.072
	small	28%	0.98	−2.01	0.08	0.68	0.87	1.33	2.52	0.63
	tiny	41%	2.69	3.82	2.18	2.17	3.10	3.58	2.16	2.81
RE-XLNet	base	15%	2.20	−0.43	−0.07	0.22	0.03	2.39	2.16	0.92
	small	21%	1.47	0.26	0.11	−0.34	0.03	3.42	4.33	1.32
	tiny	28%	1.96	3.19	0.47	−0.22	0.19	4.46	6.13	2.31
RE-BERT	base	15%	0.73	−0.54	0.48	−1.49	0.85	2.36	1.80	0.59
	small	21%	3.43	0.08	1.72	0.45	1.62	3.78	1.44	1.78
	tiny	28%	4.90	−0.94	3.48	1.49	2.66	7.65	1.80	3
RE-DistilBERT	base	15%	1.47	5.24	0.86	0.34	0.13	5.80	2.16	2.28

**Table 3:** We evaluate our compression method against different compression techniques that do not use any fine-tuning steps on the RoBERTa model (or any usage of the training data after compression). The following table reports the drop in accuracy on the MRPC task after using these compression techniques.

Compression Method		Embedding Layer Compression Rate	MRPC
SVD	base	15%	5.70
	small	28%	6.25
	tiny	41%	9.51
L1PCA [73]	base	15%	3.04
	small	28%	4.94
	tiny	41%	18.48
Pruning [74]	base	15%	1.90
	small	28%	2.17
	tiny	41%	2.98
Random pruning	base	15%	1.36
	small	28%	3.06
	tiny	41%	4.38
SynFlow [75]	base	15%	0.81
	small	28%	1.60
	tiny	41%	2.75
Our compression	base	15%	0.49
	small	28%	0.98
	tiny	41%	2.69

**Figure A1:**
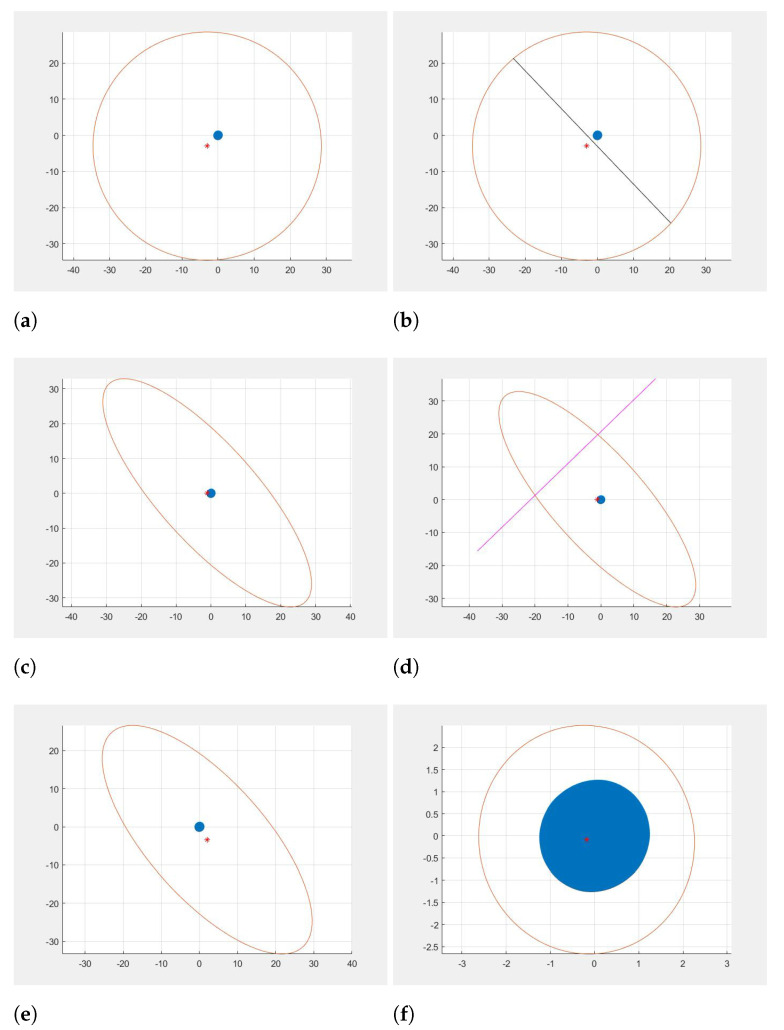
*Computing the Löwener ellipsoid*. *Step I*:We start with an ellipsoid that contains our level set (the blue body). From here, the basic ellipsoid method is invoked, i.e., while the center is not contained inside the level set (blue body), a separating hyperplane between the center of the ellipsoid and the level set is computed, and the ellipsoid is stretched in a way such that the center moves closer in distance to the level set. The basic ellipsoid method halts when the center is contained in the level set (see (**a**–**c**) for illustration of the ellipsoid method). *Step III*:We compute a contracted version of the current ellipsoid and check if all of its vertices are contained in the level set. If there exists one ellipsoid’s vertex which is not contained in the level set, we find the farthest vertex of the contracted ellipsoid from the level set and compute a separating hyperplane between it and the level set. Then, the ellipsoid is stretched such that this vertex becomes closer to the level set presented in (**d**,**e**). We then loop StepsII–III until the contracted ellipsoid’s vertices are contained in the level set (see (**f**)).

## Appendix A. Computing the Löwner Ellipsoid

For an input matrix $A\in {\mathbb{R}}^{n\times d}$ of rank *d* and a number p≥1, we now show how to compute the *Löwner ellipsoid* for the set $L:=\left\{x|x\in {\mathbb{R}}^{d},{∥Ax∥}_{p}\leq 1\right\}$. This is a crucial step towards computing the ${∥\cdot ∥}_{p}$-SVD (see Definition 1) for the matrix *A* in the context of the $\ell_{p}$-low rank approximation problem, which will allow us to suggest an approximated solution (See Theorem 5).

**Overview of Algorithm A1 (computing the Löwner ellipsoid).**Algorithm A1 receives as input a matrix $A\in {\mathbb{R}}^{n\times d}$ of rank *d* and a number p≥1. It outputs a Löwner ellipsoid for the set *L* (see Line 1 of Algorithm A1). First, at Line 1, we initialize *L* to be set of all the points *x* in ${\mathbb{R}}^{d}$ such that ${∥Ax∥}_{p}\leq 1$. At Lines 2–5, we find a ball *E* in ${\mathbb{R}}^{d}$ of radius *r*, which contains the set *L*, and its center is set to be the origin $0_{d}$. Then, we build a diagonal matrix *F*, where we set its diagonal entries to *r*.

Lines 8–12 represent the pseudo-code of the basic ellipsoid method which is described in detail in [76], where we set *H* to the separating hyperplane between *c* (the center of the ellipsoid *E*) and *L*; *b* is set to be the multiplication between *F* and the normalized subgradient of ${∥Ax∥}_{p}$ at x=c, where *b* is used to set the next candidate ellipsoid.

In Lines 13–17, we compute the next candidate ellipsoid *E*, and based on it, we set $\tilde{V}$ to be the set containing the vertices of the inscribed ellipsoid $\frac{1}{d}\left(E-c\right)+c$ in *L*. Now, if $\tilde{V}\subseteq L$, then we halt the algorithm; otherwise, we find the farthest vertex point *v* in $\tilde{V}$ from *L* with respect to ${∥Ax∥}_{p}$, and finally, we set *H* to be the separating hyperplane between *v* and *L*.

Lines 19–25 present the pseudo code of applying a shallow cut update to the ellipsoid *E*; this is described in detail in [76]. Finally, at Line 27, we set *G* to be the Cholesky decomposition of $F^{-1}$ (see [77] for more details). For formal details, see Theorem 2.

**Algorithm A1:** Löwner (*A*, *p*)
